# Eye Examination Recency among African American Older Adults with Chronic Medical Conditions

**DOI:** 10.3390/healthcare8020094

**Published:** 2020-04-12

**Authors:** Mohsen Bazargan, Tavonia Ekwegh, Sharon Cobb, Edward Adinkrah, Shervin Assari

**Affiliations:** 1 Department of Family Medicine, Charles R. Drew University of Medicine and Science (CDU), Los Angeles, CA 90059, USA; Mohsenbazargan@cdrewu.edu (M.B.); sharoncobb1@cdrewu.edu (S.C.); edwardadinkrah@cdrewu.edu (E.A.); 2 Department of Family Medicine, University of California, Los Angeles (UCLA), Los Angeles, CA 90095, USA; 3 School of Nursing, Charles R. Drew University of Medicine and Science, Los Angeles, CA 90059, USA; TavoniaEkwegh@cdrewu.edu

**Keywords:** health care use, health services, African-Americans, ethnic groups, health disparities, eye examination, underserved populations

## Abstract

*Background:* Pervasive racial and economic inequalities have a disproportionate impact on health care utilization among African Americans. One area where we see such disparities is in the recency of eye examinations among the economically disadvantaged. However, our current understanding of the barriers and facilitators of eye examinations in underserved African-American older adults is limited. *Aims:* Building on Andersen’s model of health service use and using an exploratory approach; we tested various demographic, social, and health factors that were associated with eye examination among underserved middle-aged and older adults in South Los Angeles. We examined predisposing characteristics, enabling factors, and need-for-care characteristics. *Methods:* With a cross-sectional design, we conducted this survey on a convenience sample of (*n* = 740) non-institutionalized African-American older adults who were 55+ years old and residing in South Los Angeles, CA, USA. Data were collected on demographic factors, continuity of care, access to care, self-rated health, chronic medical conditions, and depressive symptoms. The outcome was recency of eye examination. Multivariate regression was used for data analysis. *Results:* 59% of the participants had received at least one eye examination during the last 12 months. A total of 17% had an eye examination within the last two years. Notably, 26% of diabetic participants did not have an eye examination within the last two years. One out of four participants indicated that, within the last two years, no provider ever recommended that they receive an eye examination. Age, education, continuity of medical care, accessibility of medical care, satisfaction with medical care, providers’ recommendation for eye examination, self-rated health, and a diagnosis of hypertension and diabetes mellitus were predictors of eye examination recency. Overall, our analysis indicates that these enabling factors accounted for most of the variance in the recency of eye examinations. *Conclusion:* A large proportion of underserved African-American middle-aged and older adults in South Los Angeles do not comply with the recommended annual eye examination. This is, in part, because about one-third of them have not received an eye exam recommendation from their health care providers. However, a wide range of factors such as age, education, continuity of care, satisfaction with access, self-rated health, and a diagnosis of hypertension and diabetes mellitus, also influence whether or not African-American middle-aged and older adults receive an eye examination. Programs should address a wide range of multi-level factors to tackle this health inequality.

## 1. Introduction

Vision impairment among middle-aged and older adults has major consequences with considerable implications for the individuals, caregivers, health care systems, and society [1]. Numerous studies among middle-aged and older adults in the USA have established associations between visual impairment and poor quality of life [2], cognitive and functional difficulties [3,4], increased risk of falls [5], anxiety and depression [6], greater health care resource use and hospitalization [7], reduced social participation [8], increased difficulty performing activities of daily living, mobility disability [9], overall health status (both objective and subjective), and mortality [1]. Vision impairment is associated with both physical and mental illnesses, including hypertension, diabetes, stroke, kidney disease, arthritis, depression, and sleep and mood disorders [10,11]. Individuals with visual impairment may also have worse vision-related quality of life attributed to ocular diseases such as glaucoma [12], retinopathy [13], cataracts [14], vernal keratoconjunctivitis [15], and other degenerative eye diseases. Building on a well-established and widely used theoretical framework of health services utilization, this study explored the effects of predisposing factors, enabling factors, and need-for-care as determinants of eye examination recency among a sample of underserved African-American middle-aged and older adults (i.e., aged 55 years and older) in South Los Angeles, CA, USA.

Vision impairment disparities among middle-aged and older adults in the USA have been well established. The most up-to-date available data on the epidemiology of visual impairment shows that middle-aged and older African-Americans have a higher overall age-adjusted prevalence of uncorrected visual impairment and blindness than non-Hispanic whites and other racial and ethnic groups [16]. The age-adjusted prevalence of uncorrected vision impairment among middle-aged and older African-American adults and non-Hispanic whites is estimated to be 5.51% and 4.04%, respectively [17]. Furthermore, the age-adjusted prevalence of blindness among middle-aged and older African-American adults is almost twice that of non-Hispanic whites (2.48% and 1.25%, respectively) [17].

There may be existing differences in ocular characteristics among African-Americans and whites. A well-designed study conducted with 1221 African-American and white participants of European descent, shows that African-Americans have a higher prevalence of refractive errors (e.g., nearsightedness and farsightedness), thinner corneas, larger cup-to-disc ratios, and larger disc and rim areas in all diagnostic groups [18]. Causes of visual impairment among African-Americans are more attributed to cataracts and glaucoma, which are disease-related, compared to whites with age-related macular degeneration [19]. These characteristics increase the vulnerability of African-American middle-aged and older adults to vision impairment, particularly if they have diabetes or other age-related co-morbidities. Another study with 6000 African-Americans aged 40 years and older documented that African-Americans have unique demographic as well as ocular characteristics that impact both the incidence and the progression of eye diseases in this vulnerable population [20].

Despite a high risk of vision impairment [17], the frequency of eye exams among African-American middle-aged and older adults is alarmingly low [21]. Data from the National Health Interview Survey (2014–2016) suggest that racial and ethnic minorities are more likely to face difficulties using eye care [22]. Periodic eye examinations for middle-aged and older adult African-Americans, particularly those with diabetes and other co-morbidities, are extremely important. This is an issue that needs urgent attention and intervention. In addition, older African-American adults with late-life vision impairment experience multiple jeopardies related to chronic disease, not having their needs accommodated, additional disability, and health needs that are not cared for by the existing health care system [23]. Knowing the barriers to appropriate eye examination is a prerequisite for the development of a successful screening program [24].

### 1.1. Theoretical Framework 

Andersen’s health care services utilization framework [25,26,27] is a useful and widely used framework for investigating health care service utilization by middle-aged and older adults [28,29,30]. The aim of this theoretical framework is to discover potential factors that either impede or facilitate timely utilization of health services across populations through various individual, social, and contextual factors [31]. According to this model, predisposing characteristics, enabling factors, and (perceived and objective) need are potential predictors of health service utilization. In addition, there are personal health practices that are directly related to utilization of medical care and health services [27,32].

### 1.2. Aim

Building on Andersen’s model of health service use [25,26,27] and using an exploratory approach, we tested various demographic, social, and health factors associated with eye examination among underserved middle-aged and older adults in South Los Angeles. We analyzed predisposing characteristics, enabling factors, and need-for-care characteristics. 

## 2. Methods

This community-based, face-to-face cross-sectional survey included a convenience sample of 740 non-institutionalized underserved African-American adults aged 65 years and older. Participants were drawn from 11 senior housing units, 16 African-American churches, and a public housing project all located in the Service Planning Area Six (SPA 6) of Los Angeles County. Referred to as “South Los Angeles,” SPA 6 serves the communities of Athens, Florence, Compton, Crenshaw, Paramount, Lynwood, Hyde Park, and Watts, which, together, comprise a population of over one million. South Los Angeles is one of the most underserved and under-resourced service planning areas in the County of Los Angeles. Nearly 34% of the residents of SPA 6 have household incomes below the federal poverty level. One out of four adults in SPA 6 has been diagnosed with hypertension and age-adjusted coronary heart disease. SPA 6′s age-adjusted death rate due to diabetes is 37.6 per 100,000 population, which is five times larger than that of the West Los Angeles Service Planning Area (SPA 5), which has a death rate of 7.5 [33].

Eligible participants included those who (1) identified as African-American or Black, (2) were 55 years of age or older, (3) had the ability to complete an interview in English, and (4) resided in the SPA 6 area. After completing eligibility screening and providing written consent, participants were enrolled in the study. Trained research assistants traveled and conducted a 60 to 90-minute face-to-face structured interview with participants in their home. The final sample included 740 African-Americans.

The Institutional Review Board (IRB) of the Charles R. Drew University of Medicine and Science (CDU; IRB #14-12-2450-05), Los Angeles, approved this study.

### 2.1. Measurements

#### 2.1.1. Predisposing Characteristics 

Age, gender, educational attainment, and living arrangement were measured using standard instruments. Age was a continuous measure. Gender was coded as 0 for males and 1 for females. The living arrangement was a nominal variable with living alone coded as 1.

#### 2.1.2. Enabling Factors

Six factors were employed to document the enabling characteristics of our participants: (1) financial difficulty, (2) continuity of medical care, (3) accessibility of medical care, (4) satisfaction with medical care, (5) Health Maintenance Organization (HMO) membership, and (6) eye examination recommendation by a provider.

*Financial Difficulty*. Financial difficulty was measured using a five-point Likert scale varying from 1 for always to 5 for never. Participants were asked how often in the last 12 months they experienced considerable difficulty to (1) buy food, (2) buy clothes, (3) pay rent/mortgage, (4) pay monthly bills, and (5) make ends meet. A higher score was reflective of fewer financial difficulties within the last 12 months. This measure had high reliability (α = 0.934). 

*Continuity of Medical Care.* Medical care continuity was self-reported by the participants. Individuals reported their medical care continuity using the following three items: (1) type of place where they usually receive medical care, (2) whether they usually go to the same place for medical care, and (3) whether they are usually seen by the same health care provider. Responses to the first item were private doctor’s office or medical group vs. other settings. Private office or medical group was coded as 1 and any other response was coded as 0. Responses to the second and third items were coded 1 for yes and 0 for no. The total score ranged from 0 to 3, with a higher score reflecting a greater level of continuity of care. 

*Accessibility of Medical Care*. Ease of access to medical care was measured using the following three items. Participants reported how easy it is for them to (1) visit a doctor when they need medical care, (2) get a routine physical examination if they wanted one, and (3) travel to medical appointments. This variable was treated as a continuous measure with a higher score indicating less difficulty accessing care [34].

*Satisfaction with Medical Care.* Satisfaction with medical care was evaluated using the following three items. Participants were asked how satisfied they were with (1) the medical care they were currently receiving, (2) how much medical care was available for them, and (3) their access to preventative services and routine/annual checkups. A higher score indicated greater satisfaction [34]. 

*Health Maintenance Organization (HMO) Membership.* We asked participants about their health care coverage. Based on their report, we categorized individuals as HMO members or non-members, which analyzed as a dichotomous variable. HMO membership included all public insurance such as Medicare/Medicaid and private HMO insurance. Focus on HMO membership was selected due to African-Americans with HMO experiencing delays in their source of care and, consequently, going to the emergency room for care [35]. HMO members are more likely to face disparities in the utilization of healthcare specialists [36].

*Eye Examination Recommendation*. Participants were asked if a provider had recommended that they receive an eye examination. This was treated as a dichotomous variable: 0 = did not receive a recommendation, 1 = received a recommendation.

### 2.2. Need-for-Care Characteristics

Need-for-care characteristics were measured using self-rated health (including self-reported major chronic conditions and number of providers) and an instrument that captured depressive symptoms. 

*Self-Rated Health (SRH)*. A standard item was used to measure self-perceived health status. Participants were asked the following single question: “In general, would you say your health is (1) excellent, (2) very good, (3) good, (4) fair, and (5) poor?” This measure is commonly used in national surveys [37].

*Chronic Disease*. Additionally, participants were asked to report whether they had been diagnosed with any one of the following major chronic conditions: hypertension, diabetes, stroke, or a heart condition. Lastly, participants were asked to report how many different providers they had visited within the last 12 months. 

*Depressive Symptoms*. To measure the severity and frequency of depressive symptoms, the Geriatric Depression Scale (Short Form) (GDS-SF) was used. This 15 item-measure applied “yes” or “no” items [38,39]. The scale provides a total score with a range between 0 and 15, where higher scores indicate higher levels of depressive symptoms. The GDS-SF has revealed excellent validity as reliability [38,39].

## 3. Outcome

*Eye Examination Recency.* A single item was employed to determine how recently participants had received an eye examination by an ophthalmologist. Respondents were asked: “When was the last time you had an eye examination by an eye doctor?” Response categories included: 1 = less than six months ago; 2 = six months to one year ago; 3 = one to two years ago; 4 = three to four years ago; and 5 = four to five years ago; 6 = five or more years ago; and 7 = never.

### Statistical Analysis

We used SPSS (SPSS Inc., Chicago, IL, USA) for Windows to synthesize data. To describe our sample, we reported mean and standard deviation for our continuous variables, and n and percent for the categorical variables. For bivariate analysis, we used the t-independent samples test, Man Whitney, and Chi square to test the associations between eye examination and all other variables. Lastly, the multiple linear regression technique was employed to evaluate the independent impact of the predisposing, enabling, and need-for-care characteristics of participants on recency of eye examination.

## 4. Results

### 4.1. Participants

Table 1 presents descriptive statistics of our sample. The mean age of participants was 71.7 (SD: 1.36) years. The range was 55–96 years. The mean education was 12.7 years with one out of four participants having no formal education beyond eleventh grade. Slightly less than 36% reported completing high school. Over 35% of the sample reported being enrolled in an HMO. With regard to self-rated health status, only 6.2% of the sample had excellent health. A total of 36% had good health, and 31% and 7% had fair and poor health, respectively. More than 30% and 35% reported that they have been diagnosed with heart conditions and with diabetes mellitus, respectively. One out of three had visited more than one type of physician (Table 1).

### 4.2. Eye Examination

A total of 59% of participants reported that they had an eye examination within the last 12 months. Another 17% and 9% had an eye examination between 1–2 and 3–4 years ago, respectively. However, 13.4% of subjects reported no eye examination in the last five years. One out of three (32.3%) participants indicated that no provider recommended that they have an eye specialist examine their eyes. Additionally, 20% of participants with diabetic mellitus reported that they were not told by their providers that they need periodic eye examinations. Similarly, 27% of participants with a history of stroke or a heart condition claimed that their providers did not recommend an eye examination (Table 2).

### 4.3. Bivariate Correlations

Table 2 and Table 3 report the bivariate correlations between participant characteristics (predisposing, enabling, and need-for-care) and eye examination recommendation. These tables show that the delay of eye examination was associated with male gender, less education, older age, not receiving medical care from an HMO, lower levels of continuity of, and satisfaction with medical care, lower self-rated health, and not being diagnosed with diabetes or hypertension. Additionally, at the bi-variate level, those who said their providers did recommend an eye examination tended to be older, had a higher number of providers, and had diabetic mellitus.

### 4.4. Multiple Linear Regression

Table 4 shows the results of our multivariate analysis of eye examination recency. Age and education were the only predisposing characteristics significantly correlated with recency eye examination. Controlling for all other relevant variables, older participants and less educated participants were less likely to have had a recent eye examination. Among enabling characteristics, lower levels of continuity of care and satisfaction with access, availability, and quality of care were associated with non-recent eye examination. Lastly, four variables manifested the level of need-for-care associated with eye examination recency: hypertension, diabetes mellitus, self-rated health status, and eye examination recommendation. Controlling for predisposing and enabling characteristics, self-rated health was associated with more recent eye examination. Similarly, participants with hypertension and diabetes mellitus had more recent eye examinations. Lastly, controlling for all other variables, those who received an eye examination recommendation from a provider were more likely to have had a more recent eye examination. Provider recommendation (beta = −0.332, 95% CI = −1.572 to −1.030) for those with diabetes mellitus (beta = −0.147; 95% CI = −833 to −295) showed the largest b coefficients for having a recent eye examination. 

## 5. Discussion

Only 59% and 76% of participants aged 55 years and older had received an eye exam during the last 12 months and the past two years, respectively. Additionally, one out of four diabetic participants reported no eye exam within the last 12 months. We had conducted a similar study more than two decades ago among a sample of 998 underserved African-American older adults. That study showed 64% of participants (aged 65 years and older) reported receiving an eye exam within the last 12 months [23]. Twenty-two years later, we documented no improvement in frequency of eye examination. Only around 60% of African-Americans aged 65 and older had an eye exam within the last 12 months. Both of our studies (the one conducted in 1998 and the present study) echo the results of the National Health Interview Survey that clearly suggest racial/ethnic minorities are more likely to face challenges accessing and using eye care [22]. Moreover, our two studies indicate that economically disadvantaged African-American residents in SPA 6 are experiencing decades of health disparities, which may lead to greater cyclic complications and long-term disability [40]. This may infer that there is a lack of resources and supportive care for eye care among this population, which will require systemic changes at both the local and county level for this vulnerable group. This rate of eye examination among African-Americans is similar to other findings in the literature. Kamel and colleagues (2001) uncovered that African-Americans were less likely to visit an eye specialist within the previous five years compared to whites, which stands out as rather poor in contrast with the rate of eye examination in the general population. Furthermore, older African-Americans have noted that their visual impairment has resulted in functional decline, such as reading difficulties with low rates of rehabilitation referral [41]. 

Our multiple linear regression analysis (which includes all predisposing, enabling, and need-for-care characteristics) explains 20% of the variance in the recency of eye examination. We found that enabling characteristics account for most of the variance (81% of 20%) in the recency of eye exams, which include lower levels of continuity of care and satisfaction with access, availability, and quality of care. Such enabling characteristics contribute to attending eye specialists, while need-for-care is constant. Ela and Lee (2014) explored barriers to eye care among high-risk individuals, which included cost, healthcare provider-patient relationship, lack of trust, accessibility to care, and overall communication. Recommendations to improve utilization include greater awareness, lower costs, and access to insurance, which will foster trust, especially among underserved communities.

Multiple chronic illnesses (hypertension and diabetes) were revealed to be predictors for more recent eye examinations in addition to being associated as a factor for higher need-for-care. Among African-Americans, visual impairment and retinal damage may be increasingly exhibited with multiple sclerosis [42], diabetes [43,44], hypertension and related cardiac diseases [44,45], and cerebrovascular disease [44,46]. The frequency of eye exams in our sample of diabetic African-American individuals aged 55 years and older was mirrored the findings of the National Poll on Healthy Aging. Among 2013 diabetic individuals aged 50 to 80 years, 72.2% reported undergoing an eye examination in the past year and 91.3% in the past two years [47]. According to the National Health and Nutrition Examination Survey over a 10-year period, vision impairment related to diabetes increased by 20% in the US population [48]. This finding adds to the existing literature on the systemic changes and health care policies that are needed to address vision-threatening eye problems among underserved ethnic minority older adults, particularly those with diabetes mellitus and other co-morbidities that may contribute to deteriorating vision. Compared to whites, African-Americans are more likely to suffer from vision-threatening conditions, such as diabetic retinopathy [49]. Strategies to improve timely routine eye care and management should be fostered, especially for those who have multiple chronic conditions, and are at risk for vision-threatening diseases.

Provider care and recommendations for eye exams play an essential role in the promotion of vision and eye health, which was revealed to be the strongest factor for recent eye examination. Limited available studies among older African-American adults suggest that recommendations by primary care professionals and personal reminders (e.g., telephone calls) remain an important part of any intervention aimed at increasing the frequency of dilated eye examinations [50,51]. In particular, primary care providers, such as physicians, physician assistants, nurse practitioners, and clinical nurse specialists, stand on the front line in recommending routine eye exams to prevent visual impairment among older ethnic adults with diabetes and other co-morbidities. McGwin and colleagues (2010) reported that an estimated 33% of older adults in the United States did not visit an eye care provider, which partially attributes it to cost and insurance barriers [52]. An additional study by Owsley and colleagues (2006) revealed that the eye care providers held negative perceptions of older African-Americans with regard to their attitude toward eye care [53]. Furthermore, they believed African-Americans did not engage in activities to improve eye care [53]. Future research needs to implement behavioral change interventions among eye care providers toward African-Americans and potentially reduce any implicit bias. 

### 5.1. Implications for Health Policy

The US government has not yet established a national health policy regarding objectives for improving vision health. Under the Affordable Care Act (ACA), vision coverage is still not mandated for adults, which may influence primary healthcare providers in their decision-making of eye exam recommendations [54]. This may lead to vision-related disparities for underserved groups with particular predisposing characteristics (older age and lower education level). In addition, declining vision health for vulnerable populations, such as the elderly, can lead to increasing health disparities. Currently, the United States Preventative Services Task Force asserts that there is no sufficient evidence on the benefits and risks of screening for impaired visual acuity in older adults to make recommendation for or against the screening in primary care settings such as outpatient clinics and ambulatory care centers [55]. African-Americans who reside in economically disadvantaged areas, such as SPA 6, may have low access to health care, due to social stratification, health beliefs, and mistrust [56], which should be accounted for with policy development. 

Leading to delayed care and worsening vision, it is imperative that federal and state policy provide more action toward this area to prevent related symptoms or disease progression that can lead to greater morbidity and potential mortality. It is recommended that primary care healthcare providers be tasked with greater autonomy and responsibility for treating and education populations about their vision health. Primary care providers should leverage their expertise to become involved in advocating for policy and legislation change for vision health. Primary care providers can make a significant difference in health policy outcomes and the overall improvement of health equity and vision health. This can be accomplished in a variety of ways such as through (1) organized communication designed to provide accurate relevant information that establishes vision health as a national priority, (2) health promotion services that are community-centered and population-focused, (3) data analytics that convert data into actionable information to guide and inform standardized public policy, and (4) expansion of vision-related services. The following recommendations provide foundational support for sustainable populations and community-based initiatives. It opens a broad spectrum of reflection of current practices and policies that impact health equity, access to quality vision care, and helps establish new evidence-based policies and practices that will influence population health with a measurable impact on eye and vision health.

Additionally, developing programs centered around vision health and integrating community resources, especially for underserved groups, is critically needed. Targeted policy development to improve eye and vision health, through: (1) risk identification and early detection of ocular disease, (2) diagnosis of an at-risk condition for vision impairment (i.e., diabetes), and (3) appropriate follow-up and referral for eye exams and management. Stakeholders need to develop innovative health policy for eye care, which involves community engagement and social determinants, including policy makers, academia, and community members. The power of media is another effective tool that can be used more widely to influence health policy outcomes and an effective population health strategy that both enhances and promotes the awareness of vision health.

Vision impairment, which can result in adverse events, and further regression of health requires necessary focus. Further work is needed in the nation, including California, where falls among those who are visually impaired and elderly is at 59%. This may also place a major burden on the health care system, which may require further interventions for cost-effectiveness. The ability of the health care system, including providers to provide vision care, can be improved by innumerable public policy decisions.

### 5.2. Future Steps

The findings of this research indicate a definite need for coordinated efforts to expand and further develop vision management, and public health capacities in primary health care settings for African-American older adults. The American Academy of Ophthalmology as well as the American Diabetes Association both recommend annual dilated eye examinations for individuals with diabetic mellitus [57,58]. It is concerning that almost 15% of the diabetic participants in our study did not have an eye examination within the last two years. Additionally, multivariate analysis detected no connection between stroke and heart conditions and eye examination among our sample of middle-aged and older underserved African-American adults. Furthermore, it is also very important to note that 20% of our sample who were diagnosed with diabetic mellitus expressed that, within the last two years, they have not been advised by their providers on the importance of periodic eye examinations. To ensure better compliance, the combined efforts of providers, including federal, state, and local health entities is necessary. Special efforts should center on groups that are at higher risk for not receiving an eye exam recommendation or eye exam based on age, race, and the socioeconomic status (SES).

A systematic review of literature show that, while public health interventions are necessary to preserve visual acuity in racial and ethnic minority communities, further research is required to understand the risk factors and management of vision impairment. There is a need for more studies to understand why African-Americans underutilize eye care services. Research in this area would shed more light on specific factors that influence the use of eye-care among African-Americans. It is essential for health care providers to screen minority individuals with diabetes for diabetic retinopathy and implement available interventions that can reduce its impact [59]. Enhancing diabetes-related vision care among racial and ethnic minority patients can potentially decrease complications due to diabetic retinopathy. Such studies are needed to tackle related disparities, including aspects of disease management and duration [43,59]. Therefore, given the impact of racial disparities on vision screening, it is important to conduct interventional studies to motivate and train health care providers practicing in underserved communities to comply with national screening guidelines. 

The data demonstrates that, even when controlling for the eye examination recommendations, both satisfaction with and continuity of medical care remain statistically significant correlations for recency of eye examination. National data clearly show that continuity of care promotes the likelihood of clinical preventive services among older adults. This effect is particularly relevant for having a regular place of care or a regular care provider [60]. Thus, it is essential to have a preferable source of care, which works as a gatekeeper and facilitate and implement preventive medical care among underserved minority, older adults.

### 5.3. Limitations of the Current Study

Several factors limit the internal and external validity of this study. First, cross-sectional studies cannot make causal inferences. Second, no data on the health care system was collected. Third, we did not have access to objective medical histories and records for the participants. We relied on self-rated health and self-report of chronic conditions. Lastly, this study did not include details on the participants’ vision conditions.

## 6. Conclusions

The frequency of eye examinations among the sample of underserved African-Americans was alarmingly low. This requires extensive intervention, particularly for those older underserved African-Americans with diabetes mellitus and other co-morbidities. We documented that participants’ enabling characteristics (specifically, provider recommendation and continuity of and satisfaction with care) played a significant role in sending the study participants to an eye specialist. Further studies must focus on interventions that address these enabling factors and that train and motivate primary care providers serving underserved communities to adhere to national screening guidelines.

## Figures and Tables

**Table 1:** Characteristics of middle-aged underserved African-American adults (*n* = 740).

Characteristics	*n*	%
Gender		
Male	266	35.9
Female	474	64.1
Age		
55–64	120	16.2
65–74	360	48.6
≥75	260	35.1
Education		
No high school diploma	183	24.7
High school diploma	265	35.8
Some college or college degree	292	39.5
Living Alone		
No	294	39.7
Yes	446	60.3
HMO Status		
No	478	64.6
Yes	262	35.4
Stroke		
No	633	85.8
Yes	101	14.2
Diabetes		
No	478	64.8
Yes	260	35.2
Heart Conditions		
No	514	69.6
Yes	224	30.4
Hypertension		
No	62	8.4
Yes	664	91.6
		Mean ± SD
Age (years: 55–96)		71.7 ± 0.36
Education Attainment (1–16)		12.7 ± 2.24
Financial Strains (1–5)		1.8 ± 1.13
Satisfaction with Care (1: Low to 5: High)		3.4 ± 0.90
Continuity of Care (0–3)		2.49 ± 0.66
Number of Providers (1–10)		2.19 ± 1.34
Depressive Symptoms (GDS: 0–15)		2.5 ± 2.77

**Table 2:** Bi-variate correlates of eye examination and eye recommendation among underserved middle-aged and older African-American adults (*n* = 740).

Independent Variables	Eye Examination (Last 2 Years)		*p*	Eye Examination Recommended		*p*
	NO *N* (%) or (*X* ± SD)	Yes *N* (%) or (*X* ± SD)		NO *N* (%) or (*X* ± SD)	Yes *N* (%) or (*X* ± SD)	
Gender						
Male	75 (29)	188 (72)	0.040	95 (36)	168 (64)	0.099
Female	101 (22)	364 (78)		140 (30)	325 (70)	
Age						
55–64	42 (36)	76 (64)	0.002	50 (42)	68 (58)	0.013
64–96	134 (22)	476 (78)		185 (30)	425 (70)	
Education						
No High school Diploma	59 (32)	123 (68)		62 (34)	120 (66)	
High School Diploma	117 (21)	429 (79)	0.003	173 (32)	373 (68)	0.552
Financial Strains	(4.04 ± 1.21)	(4.22 ± 1.08)	0.064	(4.08 ± 1.11)	(4.23 ± 1.39)	0.115
Living Alone						
No	67 (23)	223 (77)	0.582	89 (31)	201 (69)	0.467
Yes	109 (25)	320 (75)		146 (33)	292 (67)	
Access, Availability, and Quality of Medical Care	(10.02 ± 2.23)	(10.19 ± 2.29)	0.375	(10.14 ± 2.25)	(10.15 ± 2.24)	0.968
Continuity of Medical Care	(2.36 ± 0.74)	(2.53 ± 0.63)	0.004	(2.43 ± 0.71)	(2.51 ± 0.64)	0.221
Number of Providers	(2.13 ± 1.19)	(2.20 ± 1.36)	0.555	(1.94 ± 1.11)	(2.30 ± 1.39)	0.001
Self-rated Health Status	(3.29 ± 1.04)	(3.08 ± 1.01)	0.015	(3.19 ± 1.02)	(3.10 ± 1.06)	0.246
HMO Membership						
No	126 (27)	348 (73)		159 (34)	315 (66)	
Yes	50 (20)	204 (80)	0.038	76 (30)	178 (70)	0.319
Stroke						
No	144 (23)	481(77)		208 (33)	417 (67)	
Yes	31 (31)	70 (69)	0.950	235 (27)	74 (73)	0.209
Hypertension						
No	19 (31)	43 (69)		21 (34)	41 (66)	
Yes	156 (23)	508 (77)	0.208	214 (32)	450 (68)	0.778
Diabetes						
No	143 (30)	328 (70)		183 (39)	288 (61)	
Yes	32 (13)	223 (87)	0.000	52 (20)	203 (80)	0.000
Heart Conditions						
No	123 (24)	386 (76)		176 (35)	333 (65)	
Yes	52 (24)	165 (76)	0.954	59 (27)	158 (73)	0.051
Eye Exam Recommendation						
No	104 (44)	131 (56)				
Yes	72 (15)	421 (85)	0.000	N/A	N/A	

**Table 3:** Correlations among eye examination and independent variables (*n* = 740).

Variables	1	2	3	4	5	6	7	8	9	10	11	12	13	14	15	16
1. Eye examination Recency	1.00															
2. Gender	−0.08 *	1.00														
3. Education	−0.11 **	0.13 **	1.00													
4. Age	−0.10 **	0.06	−0.02	1.00												
5. Living arrangement	0.00	0.09 *	−0.04	−0.02	1.00											
6. Financial strains	−0.05	0.07	0.10 **	0.40 **	−0.12 **	1.00										
7. HMO membership	−0.09 *	0.16 **	0.14**	−0.00	−0.10 **	−0.02	1.00									
8. Number of providers	−0.02	0.05	0.00	0.12 **	0.03	0.12 **	0.04	1.00								
9. Satisfaction with care	−0.08 *	0.01	0.06	0.07	−0.03	0.21 **	0.14 **	−0.06	1.00							
10. Continuity of care	−0.11 **	0.04	−0.00	0.27 **	0.03	0.19 **	0.02	0.03	−0.00	1.00						
11. Self-rated health	0.13 **	0.01	−0.03	−0.18 **	0.08 *	−0.26 **	0.01	0.10 **	−0.17 **	−0.03	1.00					
12. Stroke	0.05	0.01	−0.02	0.03	−0.03	−0.06	−0.05	0.04	−0.01	0.03	0.05	1.00				
13. Hypertension	−0.09 *	0.05	0.03	−0.12 **	0.01	−0.09 *	0.06	0.04	0.01	−0.05	0.05	0.08 *	1 **			
14. Diabetes	−0.23 **	0.05	−0.01	0.10 **	−0.05	0.05	0.08 *	0.12 **	−0.03	0.07	0.03	0.02	0.14 **	1.00		
15. Heart conditions	0.01	−0.05	−0.05	0.06	0.02	−0.00	−0.06	0.19 **	−0.06	0.04	0.10 **	0.12 **	0.07	0.02	1.00	
16. Eye examination recommendation	−0.36 **	0.06	0.02	0.10 *	−0.03	0.06	0.04	0.13 **	0.00	0.05	−0.04	0.05	0.01	0.19 **	0.07	1.00

* Correlation is significant at the 0.05 level (2-tailed). ** Correlation is significant at the 0.01 level (2-tailed).

**Table 4:** Multiple linear regression between explanatory variables and recency of eye examination among underserved middle-aged and older African-American adults (*n* = 740).

Independent Variables	B	Standard Error	Beta	T	95% CI for B	Significance
Gender	−0.142	0.134	−0.037	−1.057	−0.406–0.122	0.291
Education	−0.349	0.147	−0.083	−2.376	−0.637–0.061	0.018
Age	−0.446	0.202	−0.086	−2.205	−0.844–0.049	0.028
Living arrangement	−0.092	0.131	−0.025	−0.700	-0.348–0.165	0.484
Financial strains	0.115	0.067	0.068	1.728	-0.016–0.246	0.084
HMO membership	−0.139	0.137	−0.036	−1.013	-0.408–0.130	0.312
Number of providers	0.043	0.050	0.031	0.861	-0.055–0.140	0.389
Satisfaction with care	−0.059	0.029	−0.073	−2.028	−0.116–0.002	0.043
Continuity of care	−0.211	0.100	−0.075	−2.113	−0.408–0.015	0.035
Self-rated health	0.199	0.065	0.110	3.053	0.071–0.328	0.002
Stroke	0.241	0.185	0.045	1.304	−0.122–0.604	0.193
Hypertension	−0.508	0.225	−0.080	−2.263	−0.949–0.067	0.024
Diabetes	−0.564	0.137	−0.147	−4.120	−0.833–0.295	<0.001
Heart conditions	0.034	0.142	0.008	0.239	−0.245–0.312	0.811
Eye examination recommendation	−1.301	0.138	−0.332	−9.432	−1.572–1.030	<0.001
(Constant)	6.057	0.578	-	10.476	4.921–7.192	<0.001

Adjusted R-Square: 0.200. Standard Error of the Estimates: 1.635.
